# Mycotoxins in wheat cultivated in the Netherlands: results from eight years of field surveys

**DOI:** 10.1007/s12550-021-00427-x

**Published:** 2021-03-30

**Authors:** H.J. Van der Fels-Klerx, Marlous Focker, Theo De Rijk, Cheng Liu

**Affiliations:** 1 Wageningen Food Safety Research, Akkermaalsbos 2, Wageningen, 6708 WB The Netherlands; 2 Business Economics Group, Hollandseweg 1, Wageningen, 6706 KN The Netherlands

**Keywords:** Agronomics, Pre-harvest, Grain, Deoxynivalenol, Zearalenone, Nivalenol

## Abstract

In the period 2009–2018, an annual field survey with commercial arable farms in the Netherlands was held, to collect data on agronomics of wheat fields as well as mycotoxin concentrations of the specific wheat field at harvest. In total, 293 full farm field records over 8 years were obtained. This study aimed to investigate (i) the occurrence of deoxynivalenol (DON) and other mycotoxins, as well as correlations between these mycotoxins, and (ii) the relationships between agronomics and the DON concentration in wheat kernels for wheat cultivated in the Netherlands. Results showed that mycotoxins most frequently observed in concentrations above the limit of quantification were DON, enniatin B and B_1_, HT-2 toxin, zearalenone (ZEN) and nivalenol. On average, DON was detected in 54% of the samples (> 50 µg/kg) ranging from 19 to 92% depending on the year. Positive samples (> 50 µg/kg) had DON concentrations ranging 53–15,400 µg/kg, with a median of 228 µg/kg. Co-occurrence between DON and ZEN as well as between each of DON and ZEN with their modified forms was confirmed by the data of this study. The year influenced the DON concentration in wheat the most, followed by the region. The results of this study show that DON levels in wheat can only be influenced in a limited manner by agronomic practices such as the use of fungicides against *Fusarium* spp. around flowering, crop rotation, or the use of resistant wheat cultivars.

## Introduction

In 2018, common wheat and spelt represented 44% of all cereal grains harvested in Europe with a total production of 129 million tonnes (Eurostat 2019). Wheat is used for human and animal consumption, as well as for the production of other products (e.g. starch and bioethanol). In order to maintain a high wheat yield, disease prevention is crucial. One of the common diseases in wheat in Europe is *Fusarium* spp. infection, leading to *Fusarium* head blight (FHB) followed by the production of mycotoxins, of which deoxynivalenol (DON) is the most prominent one (Osborne and Stein 2007). *F. graminearum* and *F. culmorum* are the most common fungi species that cause FHB and produce DON in Europe (Pasquali et al. 2016). Wheat infected with FHB is often of lower quality and leads to a lower yield, leading to lower total sales price for the harvested grain (Miller 2008). Furthermore, the presence of high levels of DON can potentially lead to negative health impacts such as vomiting, diarrhoea and abdominal pain in human and reduced feed intake, weight loss and reproductive disorders in pigs and other animals (Maresca 2013). In addition to DON, *Fusarium* spp. can form other mycotoxins as well, such as nivalenol (NIV), HT-2 and T-2 toxins, zearalenone (ZEN) and fumonisins.

It has often been reported that the presence of *Fusarium* spp. and related mycotoxin production depends, amongst others, on agronomic practices like crop rotation, the soil cultivation, the susceptibility of the cultivar and the use of fungicides against FHB (Beyer et al. 2006; Janssen et al. 2019; Mielniczuk and Skwaryło-Bednarz 2020; van der Fels-Klerx et al. 2010; Zorn et al. 2017). *Fusarium* spp. infections and DON are frequently seen on wheat when it is cultivated after maize or wheat together with minimum tillage of the soil, leaving organic residues from the previous crop (Birr et al. 2019; Mielniczuk and Skwaryło-Bednarz 2020). However, effects of specific agronomic measures might vary between countries.

The first objective of this study was to evaluate the occurrence of DON and other mycotoxins as well as correlations between the different mycotoxins in wheat produced in the Netherlands. The second aims were to describe the agronomic practices, such as soil cultivation and crop rotation, used by Dutch farmers, and to investigate the relationships between the DON concentration in wheat at harvest and these agronomic practices. If such relationships between the DON concentration and agronomic practices exist, results could be used to advise farmers on agronomic practices to limit the presence of mycotoxins in wheat.

## Materials and methods

### Questionnaires and sample collection

Annual field surveys were performed in the Netherlands between 2009 and 2018, except for 2012 and 2014, using three rounds of farm questionnaires every study year. Eight years of data were therefore available and analysed in this study. Questionnaires were predefined early 2009 and were identical over the entire study period. The first questionnaire was sent to farmers in the beginning of the year asking them if they were cultivating winter wheat and, if yes, if they were willing to participate in the field survey that year. If so, the farmers were invited to answer questions about their farm location by providing their geographical coordinates, and about the agronomics of one particular wheat field, chosen by the farmer, including the winter wheat cultivar, the resistance score of this cultivar against *Fusarium* ssp., the previous crop cultivated on the field, soil type, soil cultivation and fungicides applications against FHB around flowering. The second questionnaire was sent to the participants around the wheat flowering period, with questions about the wheat flowering date, applied fungicides against FHB and the expected harvest date. A third questionnaire was sent around harvest time to ask the farmers about the real harvest date of the wheat field and whether the harvest date was delayed and, if so, why. Together with the third questionnaire and a sample collection instruction, the famers were asked to send a sample of approximately 1 kg of wheat kernels collected from the combine at harvest of the field. Farmers were asked to compose this total sample from five different subsamples (of about 200 g). The materials to collect the sample, such as plastic bags and envelopes, were provided.

## Chemical analysis of the samples

Wheat samples were sent to the laboratory of Wageningen Food Safety Research. After receival, they were directly ground and stored at −20 °C until further analysis. Samples were analysed for the presence of multiple mycotoxins using a validated and accredited analytical method similar to the method as described by López et al. (2016). Briefly, the method consisted of an acetonitrile/water extraction (QuEChERS-procedure), followed by high-performance liquid chromatography separation and triple quad mass spectrometry detection in the MRM mode. Quantification was performed by a standard addition protocol. Quality control samples were prepared by addition of known amounts of the investigated mycotoxins to blank sample material and complied to performance criteria as set in Commission Regulation (EC) No 401/2006 (Annex II). For compounds not mentioned in Annex II of Commission Regulation (EC) No 401/2006, performance criteria for recovery were set at 70–120% and repeatability (RSD_r_) at ≤ 20%. The mycotoxins analysed varied per year, as well as their level of quantification (LOQ). The reason for the latter was that the LOQ was determined each year. DON and its derivate 15-acetylDON (15-AcDON) were analysed each year. Aflatoxin B_1_, B_2_, G_1_, G_2_, agroclavine, alternariol, alternariol-methyl ether (AOH), beauvericin (BEA), diacetoxyscirpenol, fumonisin B_1_, B_2_, B_3_, mycophenolic acid, nivalenol (NIV), penicillic acid A, roquefortine C, sterigmatocystin-, T-2 toxin, HT-2 toxin, zearalenone (ZEN), α-zearalenol (α-ZEL) and β-zearalenol (β-ZEL) were analysed in all years except for 2015. Citrinin, DON-3-glucoside (DON-3-G), moniliformin (MON), nitropropionic acid and ochratoxin A were analysed for 6 years. Enniatin A, A1, B, B1 and 3-acetyl-DON (3-AcDON) were analysed for 4 years.

## Data processing

To overcome the variability due to different limits of quantification (LOQ) per mycotoxin over the study years, samples that had a concentration below the respective LOQ were set at 50% of the highest LOQ for the particular mycotoxin from all years combined. Samples with concentrations above 50% of the highest LOQ from all years combined were classified as positive samples. For 1 year, the LOQ was below 50% of the highest LOQ, meaning that samples with concentrations between this level and the LOQ of that particular year were consequently classified as negative even though mycotoxins were detected in these samples. This was, however, the case for only a few samples (related to DON). For each mycotoxin, the percentage of wheat samples having concentrations above 50% of the highest LOQ used in the analyses of particular mycotoxin in the entire study period was reported.

The agronomic information given by the farmers to each question was processed by grouping the answers into categories. The previous crops reported in the questionnaires were grouped into the following three categories: vegetables (including potatoes, onions and other vegetables), cereals (wheat, barley or maize) and others (sugar beet, pasture, flowers or a mix of different crops). For the use of fungicides against FHB around flowering, the categories ‘yes’ or ‘no’ were considered. A similar classification was used for soil cultivation: deep ploughing or minimal tillage. Tillage depth was expected to influence the presence of mycotoxin-producing fungi. Deep ploughing methods were expected to destroy and bury crop debris. Minimum tillage methods were expected to leave some crop residues on the soil surface (Edwards and Jennings 2018). For the resistance of the wheat cultivar, a score from 1 to 10 according to the Dutch system was used. The resistance scores reported in the questionnaires ranged from 5.5 (susceptible) to 8 (resistant) in steps of 0.5. These scores were grouped into two categories: crops with a resistance score between 5.5 and 6.5 and crops with a resistance score between 7 and 8. The location of the wheat field was divided into 4 regions: the North, the Middle, the West and the South-East of the Netherlands. The North consisting of the provinces Friesland and Groningen, the Middle of the provinces Drenthe, Flevoland and Overijssel, the South-East of the provinces Gelderland, Noord-Brabant and Limburg, and the West of the provinces Noord-Holland, Zuid-Holland and Zeeland. In August, when most wheat is harvested, average temperatures range from 21 °C in the North and West to 24 °C in the South-East. Most precipitation is observed in the West, followed by the North, the Middle and the South-East (KNMI 2020). Locations within each region were expected to have experienced similar weather during the growing seasons.

## Relationship agronomics and mycotoxin concentration

To investigate possible relationships between the agronomic variables and the concentration of DON in the wheat kernel samples, the DON concentrations were transformed to the natural logarithm. Possible correlations between the presence of most frequently observed mycotoxins and their metabolites were investigated with Spearman’s correlation scores, using a significance level of 0.05. The differences between DON concentrations per year, per agronomic practice used and per growing area in the Netherlands were investigated using boxplots. An analysis of variance (ANOVA) was performed to analyse the effects of the year, region and agronomic practices on the DON concentration. Due to our unbalanced dataset, type II sum of squares were applied. The residuals were assumed to be normally distributed. The following (agronomic) variables were considered: the year, the region, the previous crop, the resistance class of the cultivar, the use of fungicides against FHB and the soil cultivation. As described by Edwards and Jennings (2018), the interaction effects between agronomic practices were included if there was a biological reason behind the possible interaction effect. Since year and region effects are both (partly) related to weather, the interaction between the year and the region was included. Furthermore, the previous crop and the soil cultivation both influence the crop debris, which is an important parameter in the epidemiology of FHB. Therefore, the interaction between the previous crop and the soil cultivation was included. For all statistical analyses, the statistical software R, version 3.5.0, was used.

## Results

### The presence of mycotoxins in Dutch wheat fields

Over the eight study years, in total, 293 full farm records with wheat samples collected at harvest were obtained. The number of wheat fields varied from 17 to 86 per year. Mycotoxins that were most frequently observed to be present (in concentrations above the highest LOQ of all years combined, per toxin) were DON, enniatin B and B_1_, HT-2 toxin, ZEN and NIV (Table 1). Other mycotoxins that were detected to be present above the highest LOQ were DON-3-G, 15- and 3-AcDON, α-zearalenol and β-zearalenol, alternariol, beauvericin, enniatin A1, moniliformin, nitropropionic acid and sterigmatocystin (Table 1). Mycotoxins that were analysed but not detected above the highest LOQ in the entire study period were aflatoxins B_1_, B_2_, G_1_ and G_2_, agroclavine, alternariol-methyl ether, citrinin, diacetoxyscirpenol, enniatin A, fumonisins B_1_, B_2_ and B_3_, ochratoxin A, penicillic acid, roquefortine C, mycophenolic acid and T-2 toxin.

**Table 1:** Percentage of samples above the LOQ in wheat at harvest in the Netherlands in the period 2009–2018

	LOQ	2009 (%)	2010 (%)	2011 (%)	2013 (%)	2015 (%)	2016 (%)	2017 (%)	2018 (%)
15-acetyl-DON	25	1.6	0.0	6.7	8.0	/	0.0	0.0	0.0
3-acetyl-DON	40	0.0	/	/	8.0	/	0.0	0.0	0.0
Alternariol	10	4.8	0.0	6.7	0.0	/	0.0	0.0	0.0
Beauvericin	12.5	3.2	0.0	3.3	20.0	/	0.0	0.0	0.0
Deoxynivalenol	50	63.5	20.0	83.3	92.0	26.3	72.0	75.0	47.1
DON-3-glucoside	125	/	/	40.0	4.0	1.6	4.0	4.2	0.0
Enniatin A_1_	25	/	/	/	36.0	/	4.0	0.0	5.9
Enniatin B	25	/	/	/	92.0	/	76.0	16.7	23.5
Enniatin B_1_	25	/	/	/	48.0	/	36.0	4.2	17.6
Moniliformin	62.5	1.6	0.0	36.7	/	/	0.0	0.0	0.0
Nitropropionic acid	25	3.2	/	6.7	4.0	/	8.0	0.0	0.0
Nivalenol	200	4.8	4.0	3.3	4.0	/	8.0	4.2	0.0
Sterigmatocystin	0.5	/	0.0	0.0	0.0	/	0.0	4.2	0.0
HT-2 toxin	10	14.3	24.0	0.0	12.0	/	8.0	4.2	17.6
Zearalenone	25	4.8	8.0	70.0	20.0	/	8.0	4.2	0.0
α-Zearalenol	25	0.0	0.0	10.0	0.0	/	0.0	0.0	0.0
β-Zearalenol	25	4.8	0.0	3.3	0.0	/	0.0	0.0	0.0

“/” not analysed in the particular year

The most frequently detected mycotoxins in wheat and their metabolites were correlated pairwise (Table 2). The presence of DON and DON-3-G was highly correlated with a Spearman’s correlation factor of 0.94. The presence of DON and ZEN was correlated with a factor of 0.89. Furthermore, the presence of ZEN and α-zearalenol was correlated with a factor 0.76. The presence of enniatin B was correlated with DON (0.54) and 15-AcDON (0.52). The presence of enniatin B_1_ was correlated with 15-AcDON (0.55). The presence of NIV and beauvericin was correlated with a factor 0.74.

**Table 2:** Spearman’s correlation matrix for the most frequently detected mycotoxins in wheat in the Netherlands, in the period 2009–2018. Only the significant correlations (*p* < 0.05) are shown

	DON	DON-3-G	15-AcDON	ZEN	α-ZEL	β-ZEL	NIV	BEA	MON	AOH	HT-2	ENN A_1_	ENN B	ENN B_1_
DON	1	0.94	0.64	0.89	0.56	0.19	0.20	0.06	0.31			0.48	0.54	0.46
DON-3-G		1	0.51	0.90	0.72	0.59	0.24		0.26			0.18		
15-AcDON			1	0.43	0.11	0.29	0.39		0.08			0.71	0.52	0.55
ZEN				1	0.76		0.12		0.32		-0.05			
α-ZEL					1	0.20			0.30					
β-ZEL						1								
NIV							1	0.74				0.02	0.39	0.41
BEA								1						
MON									1					
AOH										1				
HT-2											1			
ENN A_1_												1	0.42	0.63
ENN B													1	0.84
ENN B_1_														1

**Fig. 1:**
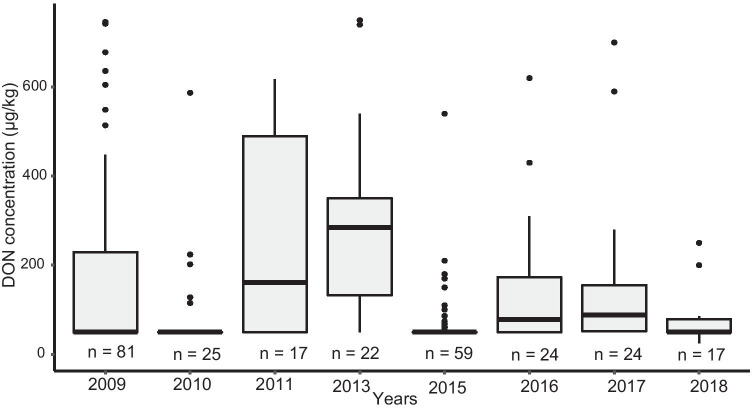
DON concentrations in wheat grains from agricultural fields in the Netherlands in the period 2009–2018: average with deviations per study year. The boxes display the first quantiles, the median and the third quantile; the whiskers display the minimum and maximum values; the dots represent the outliers

**Fig. 2:**
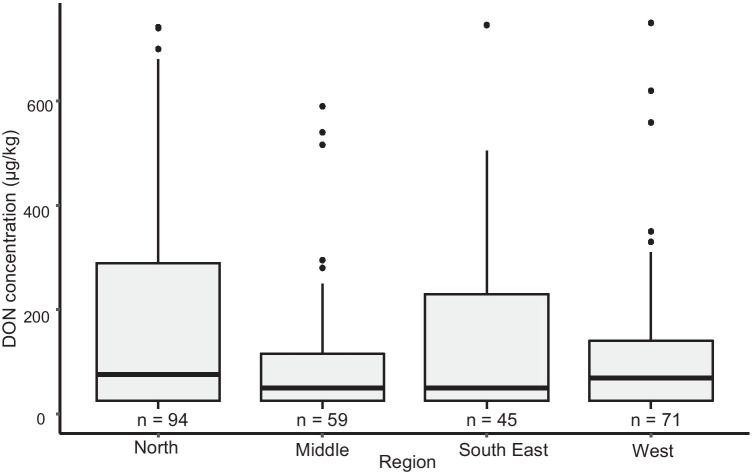
DON concentrations in wheat grains from agricultural fields in the Netherlands in the period 2009–2018: average with deviations per region. The boxes display the first quantiles, the median and the third quantile; the whiskers display the minimum and maximum values; the dots represent the outliers

The average occurrence of DON in wheat in the period 2009–2018 was 54%, ranging from 20 to 92% between the 8 study years. The positive samples (> 50 µg/kg) had DON concentrations up to 15,400 µg/kg, with a median of 228 µg/kg (Fig. 1). In 2011, high DON contamination was observed: 25 out of the 30 samples analysed contained DON concentrations above 50 µg/kg, of which 11 samples (35%) contained DON concentrations above the EU legal limit for unprocessed wheat (> 1,250 µg/kg) (EC 2006c), and 3 samples (10%) contained DON concentrations above the guidance level for feed grains of 8,000 µg/kg (EC 2006a). Furthermore, high ZEN contamination was observed in 2011 as well: 17 out of the 30 samples had a ZEN concentration above 25 µg/kg, 14 out of the 30 samples (47%) had ZEN concentrations above 100 µg/kg, the EU legal limit for ZEN for unprocessed wheat destined as food and 1 sample (3%) had a ZEN concentration above 200 µg/kg, the guidance level for feed grains. Samples with DON and/or ZEN concentrations above the guidance limits for feed were observed in 2011 only. On average, higher DON concentration was observed in the North of the Netherlands (Fig. 2), including the provinces Friesland and Groningen.

## Agronomic practices

In the last decade, the management choices of wheat farmers in the Netherlands varied and, in general, agronomic management changed towards a more sustainable farming system (no ploughing or no fungicide use) in the recent study years (Table 3). Since 2015, the use of deep ploughing decreased over the years, down to 47% in 2018 (Table 3). The use of fungicides was strongly year-dependent: for some years (2013, 2015 and 2016), almost all farmers used fungicides against FHB around flowering, whereas in other years (2010, 2011, 2018), less than one third of the farmers used fungicides. High DON levels, in combination with a low fungicide use (21%), were observed in 2011. In the following years, the use of fungicides was much higher (with 88% in 2013). The choice of more resistant cultivars was not always in favour, although a slight increase to more than half of the farmers in the study chosen resistant cultivars was observed since 2017. From 2015 onwards, cereals were used less often as pre-crop to the wheat field and replaced by vegetables, which were used more often as pre-crop. In the North and South East, farmers used cereals as previous crop more frequently and used less resistant wheat cultivars as compared with the Middle and West. In the South East, the use of fungicides around flowering was less than in the other three regions (Table 4).

**Table 3:** Agronomic practices used by wheat farmers in the Netherlands in the period 2009–2018

		2009	2010	2011	2013	2015	2016	2017	2018	Average
Soil cultivation	Deep ploughing (%)	67	80	69	80	58	56	54	47	64
	Minimum tillage (%)	33	20	31	20	42	44	46	53	36
Fungicide use	Yes (%)	51	25	21	88	98	96	62	31	59
	No (%)	49	75	79	12	2	4	38	69	41
Cultivar resistance	5.5–6.5 (%)	36	52	76	72	40	58	42	44	51
	7–8 (%)	64	48	24	28	60	42	58	56	49
Previous crop	Cereals (%)	28	52	45	48	20	12	8	18	29
	Vegetables (%)	51	40	28	28	58	88	79	76	56
	Other (%)	21	8	27	24	22	0	13	6	15

**Table 4:** Agronomic practices used by wheat farmers in the Netherlands in the period 2008–2019, per region

		Middle	North	South East	West
Soil cultivation	Deep ploughing (%)	45	54	47	49
	Minimum tillage (%)	55	46	53	51
Fungicide use	Yes (%)	69	62	44	70
	No (%)	31	38	56	30
Cultivar resistance	5.5–6.5 (%)	48	24	33	42
	7–8 (%)	52	76	67	58
Previous crop	Cereals (%)	10	38	40	22
	Vegetables (%)	71	48	35	62
	Other (%)	19	14	25	16

## Relationships of agronomic practices and DON concentration

**Fig. 3:**
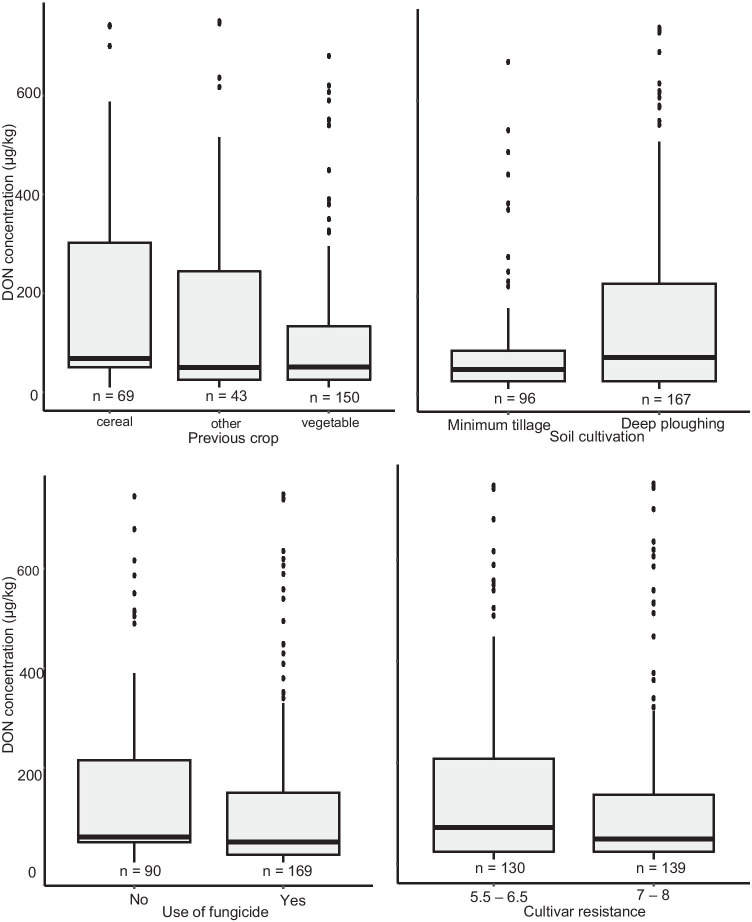
Distribution of DON concentration in the period 2009–2018 depending on the previous crop, the soil cultivation, the use of fungicides and the cultivar resistance level. The boxes display the first quantiles, the median and the third quantile; the whiskers display the minimum and maximum values; the dots represent the outliers

The relationships between the DON concentrations and the previous crop, soil cultivation, use of fungicides against FHB around flowering and the resistance class of the wheat cultivar were further analysed. Wheat fields with vegetables as the previous crop, minimum tillage, use fungicide and the use of more resistant cultivar showed a lower average DON concentration as compared with wheat fields with cereals as previous crop, ploughing and the use of less resistant cultivars (Fig. 3). In this study, the year had the most significant effect on the DON concentration in wheat (Table 5). Thereafter, the region and the interaction between the year and the region had significant effects on the DON concentration in wheat (*p* < 0.05) (Table 5). The majority of the variance in DON concentration was explained by the year (28.5%). All agronomic practices together (excluding the year and region) explained only 2.5% of the variance (Table 5). Without the variables year and region in the model, the use of fungicides around flowering, the previous crop and the resistance of the wheat cultivar had a significant effect on the DON concentration (Table 6).

**Table 5:** ANOVA results: effects of year, region, previous crop, cultivar resistance, use of fungicides against FHB and soil cultivation on DON concentrations in wheat in the Netherlands (*n* = 293), (* <0.05)

	Degrees of freedom	*F* value	*P* value	% variance explained
Year	7	17.37	0.0000*	28.5
Region	3	10.61	0.0000*	7.5
Year and region	19	1.91	0.0144*	8.5
Previous crop	2	1.37	0.2552	0.6
Soil cultivation	1	2.98	0.0853	0.7
Previous crop and soil cultivation	2	0.51	0.6016	0.2
Use of fungicides	1	0.83	0.3636	0.2
Cultivar resistance	3	1.18	0.3180	0.8
Residuals	226			53.0

**Table 6:** ANOVA results: effects previous crop, cultivar resistance, use of fungicides against FHB and soil cultivation on DON concentrations in wheat in the Netherlands (*n* = 293), (*<0.05)

	Degrees of freedom	*F* value	*P* value	% variance explained
Previous crop	2	4.29	0.0147*	3.2
Soil cultivation	1	2.31	0.1297	0.9
Previous crop and soil cultivation	2	0.22	0.8010	0.2
Use of fungicides	1	9.11	0.0028*	3.4
Cultivar resistance	3	2.76	0.0428*	3.1
Residuals	255			94.0

## Discussion

Mycotoxins that were most frequently observed above the LOQ in the current study include DON, ZEN, NIV, enniatins B and B_1_ and HT-2 toxin. DON was detected above 50 µg/kg in 54% of the samples during the eight study years. This finding is in line with a previous study in North-West Europe (Van der Fels-Klerx et al. 2012). For all years combined, this latter study reported an overall DON occurrence of 46% in wheat, maize and oats in Finland, Sweden, Norway and the Netherlands, with a median concentration of DON in winter wheat in the Netherlands of 220 µg/kg. In the UK, wheat samples were collected between 2006 and 2013 by Edwards and Jennings (2018) who reported an average DON occurrence of 79% with a median concentration of 261 µg/kg and 4% of the samples containing DON concentrations above 1,250 µg/kg. In soft wheat fields, grown between 2009 and 2011 in Italy, the occurrence of DON was 80–90% (Bertuzzi et al. 2014). Lee and Ryu (2017) performed a literature review bringing together all studies that reported on the occurrence of mycotoxins worldwide. They came up with a DON occurrence of 68% in raw wheat in Europe with a concentration range of 0.6–6,460 µg/kg.

Grains are usually contaminated with multiple mycotoxins at the same time. This study showed that the presence of DON and its modified forms such as DON-3-G in wheat were highly correlated. Likewise, the presence of ZEN and its modified forms such as α-zearalenol and β-zearalenol were correlated. Furthermore, the presence of DON and ZEN were correlated in this current study. Lindblad et al. (2013) found no correlation between DON and ZEN in Swedish wheat. In soft wheat that was grown between 2009 and 2011 in Italy, ZEN was rarely found, but was mostly found in samples showing high DON contamination (Bertuzzi et al. 2014). Edwards (2009) showed a significant relationship between DON and ZEN concentrations in wheat samples collected in the UK in the period 2001–2005. However, this study also reported on samples with high DON concentrations and low ZEN concentrations, and vice versa, showing that the correlation was not perfect. Birr et al. (2020) found higher ZEN concentrations in years with high DON concentrations during 5 years of field surveys in Germany. With respect to the presence of DON and NIV in wheat, our results on the lack of correlation between these two toxins are very consistent with previous studies (Edwards 2009; Lindblad et al. 2013; Van der Fels-Klerx et al. 2012; Vogelgsang et al. 2019). We found a lower occurrence and level of contamination for NIV than for DON in wheat. In the years 2000 and 2001, in the Netherlands, *F. graminearum* was the most predominant species responsible for FHB and the production of mycotoxins (Waalwijk et al. 2003). This is in line with more recent field surveys, held in Germany (Birr et al. 2020), Poland (Bilska et al. 2018) and Italy (Covarelli et al. 2015). NIV and DON, both type B trichothecenes, are produced by different chemotypes of *F. graminearum* (Bryła et al. 2019; Edwards 2009; Waalwijk et al. 2003). DON chemotypes were predominant in the Netherlands (Waalwijk et al. 2003). These chemotypes produce NIV as a low-level co-contaminant (Edwards 2009; Waalwijk et al. 2003). NIV is a common contaminant in wheat infected by *F. poae*. *F. poae* is, however, not a predominant species in the Netherlands (Schothorst and van Egmond 2004).

HT-2 toxin was detected in 11.3% of the samples (> 10 µg/kg) throughout the years. Edwards and Jennings (2018) reported an overall occurrence of 1.4% for both HT-2 toxin and T-2 toxin in wheat in the UK. Lindblad et al. (2013) only found HT-2 toxin or T-2 toxin to be present in three samples of Swedish spring wheat. Vogelsang et al. (2019) reported the presence of HT-2 toxin in only 1.2% of the wheat samples collected in Belgium during 8 years. Enniatins have not frequently been included in long-term field surveys. In our study, enniatins A_1_, B and B_1_ were detected in 14.2%, 46.6% and 26.2% of the samples, respectively. Decleer et al. (2019) measured enniatin A in 49% of the samples and each of enniatin A_1_, B, B_1_ in 100% the wheat samples collected in 2016 in Belgium. Orlando et al. (2019) reported an occurrence of enniatins ranging from 53% (2014) to 91% (2012) in French small grain cereal samples Overall, most of the farmers used deep ploughing as soil cultivation and rotated wheat cultivation with vegetables, in particular potatoes, or sugar beet. However, farmers in the North of the Netherlands tended to grow cereals during multiple years after each other, whereas farmers in the Middle and West rotated their wheat fields more frequently with vegetables. When cereals were used as previous crop, ploughing was more frequently used than when vegetables were cultivated the year before. The use of fungicides against FHB around wheat flowering varied a lot between years: 21% of the farmers used fungicides in 2011 whereas 98% of the farmers used fungicides in 2015. There seems to be a trend towards less deep ploughing of the fields in the last years of the study period. There also seems to be a trend towards the use of fungicides during wheat flowering in the year that follows a year with high overall DON concentrations. Our results show that most wheat farmers used a combination of several pre-harvest agronomic measures. These results comply with the results of Janssen et al. (2019) who performed a questionnaire-based study on farm management to reduce *Fusarium* spp. and related mycotoxins in the Netherlands in the year 2017. In their study, pre-harvest measures against FHB and related mycotoxins mostly applied by Dutch farmers included the use of decontaminated seeds to avoid initial fungal contamination, crop rotation, ploughing, use of a resistant wheat cultivar and the use of fungicides against FHB. Decontamination of the seeds was not considered in our study.

The second objective of this study was to investigate the relationship of the DON concentrations in the wheat samples and agronomic practices related to the specific wheat field the samples were collected from. However, our results showed that the effect of the year on DON contamination was much larger than the effects of the agronomic practices, which can be influenced by the farmers such as the previous crop, the use of fungicides against FHB around flowering, the soil cultivation and the resistance of the wheat cultivar against *Fusarium* spp. Agronomic factors together explained only 2.5% of the variance in DON concentrations, whereas the year explained 28.5% of the variance. Similar results were found by Edwards and Jennings (2018) who described the impact of agronomic factors on DON concentrations in wheat in the UK and found that the fitted model described 74% of the observed variance, of which 59% of the variance was accounted for by year and only 5% of the variance was accounted for by the agronomic factors. A Belgian field survey containing 9 years of data from experimental wheat fields showed a much higher effect of agronomic practices: the year accounted for 31% of the total variance in DON contamination, the location within Belgium for 6%, the previous crop for 5% and the wheat variety for 8% (Landschoot et al. 2012). The same trend was found in study held in 2009–2013 in Italy (Bertuzzi et al. 2014; Covarelli et al. 2015; Scala et al. 2016), and another one between 2013 and 2017 in Germany (Birr et al. 2020).

Even though the effects of agronomic factors on DON concentrations are much smaller than the effects of the year and the region, on average, lower DON concentrations were seen with vegetables as previous crop and the use of fungicides against *Fusarium* spp. around wheat flowering and the use of resistant wheat cultivars. These observations are in line with previous research (Beyer et al. 2006). Deep ploughing is expected to remove seed and crop residues that can be substrates for fungal growth and could consequently infect the next crops cultivated in the same field. The European Commission recommends to plough fields whenever possible and use minimum tillage practices in areas vulnerable to erosion (EC 2006b). However, conflicting results are seen for the effect of ploughing on DON contamination of wheat. The results of this study show that deep ploughing, on average, does not lead to lower DON levels whereas Edwards and Jennings (2018) identified minimum tillage as a great risk factor for the development of *Fusarium* mycotoxins.

High DON concentrations were observed in wheat in the years 2009, 2011 and 2013, especially in 2011, in the Netherlands. The weather conditions in 2011—with a wet and cold summer—were conducive for *Fusarium* spp. infection of the wheat. However, in this year, fungicide use during wheat flowering was low (79% did not spray), less resistant cultivars were used (only 24% of the farmers used cultivars with resistance ≥ 7) and 45% of the farmers used cereals as previous crop. Given the variation between years in the use good agronomic practices, some farmers can further improve their management to reduce *Fusarium* spp. in wheat and related mycotoxins.

To conclude, DON is the most frequently observed mycotoxin in Dutch wheat. Since wheat is an important staple crop for feed and food production, controlling mycotoxins, and DON is particular, is crucial. The results of this study show that DON levels in wheat can be influenced by agronomic practices such as crop rotation, the use of fungicides against *Fusarium* spp. around flowering and the use of resistant wheat cultivars, though too limited extent.

## Data Availability

Farm specific data and related mycotoxin data from this study are not publicly available since in agreement with the participating farmers, published results should not be traceable to the individual farmers. Aggregated data is provided in the manuscript.

## Code availability

For all analyses, the statistical software R, version 3.5.0, was used. The custom code for the statistical analysis in this study is not publicly available since the raw data cannot be shared.
